# Scalability Issues for Remote Sensing Infrastructure: A Case Study

**DOI:** 10.3390/s17050994

**Published:** 2017-04-29

**Authors:** Yang Liu, Sean Picard, Carey Williamson

**Affiliations:** Department of Computer Science, University of Calgary, Calgary, AB T2N 1N4, Canada; liuyang1520@gmail.com (Y.L.); sean@picard.ws (S.P.)

**Keywords:** remote sensing, sensor web, scientific web site, network traffic measurement, workload characterization, benchmarking, performance

## Abstract

For the past decade, a team of University of Calgary researchers has operated a large “sensor Web” to collect, analyze, and share scientific data from remote measurement instruments across northern Canada. This sensor Web receives real-time data streams from over a thousand Internet-connected sensors, with a particular emphasis on environmental data (e.g., space weather, auroral phenomena, atmospheric imaging). Through research collaborations, we had the opportunity to evaluate the performance and scalability of their remote sensing infrastructure. This article reports the lessons learned from our study, which considered both data collection and data dissemination aspects of their system. On the data collection front, we used benchmarking techniques to identify and fix a performance bottleneck in the system’s memory management for TCP data streams, while also improving system efficiency on multi-core architectures. On the data dissemination front, we used passive and active network traffic measurements to identify and reduce excessive network traffic from the Web robots and JavaScript techniques used for data sharing. While our results are from one specific sensor Web system, the lessons learned may apply to other scientific Web sites with remote sensing infrastructure.

## 1. Introduction

The field of computer systems performance evaluation [1,2,3] offers a suite of techniques to assess and improve the performance of computer systems, including network-based systems. These techniques include mathematical modeling and analysis, simulation, and experimental evaluation approaches. These techniques have been widely used over the years in many different application domains, including computer architecture [4], Internet protocols [5], queueing systems [6,7], and Web performance [8].

In this paper, we apply experimental methods from performance evaluation to a Web-based system used for collecting and sharing data from a remote sensing platform. In particular, we use active benchmarking techniques to assess the performance limits of the data collection infrastructure, and passive measurement techniques to characterize the workload of the data dissemination infrastructure. In both experiments, we identify performance issues that limit the scalability of the remote sensing system, and use the insights from our experiments to propose and evaluate improvements to the system. Similar measurement-based approaches have been used, for example, to assess the scalability and performance of Web sites for e-commerce [9], education [10], and sports [11], as well as for Internet-based video streaming services [12,13,14].

The underlying motivation for our work is the growing use of “open data” Web sites by scientific and governmental organizations. For example, an emerging trend with research funding agencies and public-funded universities is toward open access publishing and open data repositories. These publicly-accessible data repositories enable not only the sharing of scientific data among researchers world-wide, but also enable a wide variety of “citizen science” projects and outreach activities. For example, Stanford University provides large network dataset collections to the public via the Stanford Network Analysis Project (SNAP) [15], giving computer scientists, sociologists, and psychologists opportunities to test their methodologies as well as their conjectures.

At our institution, a research group in the Department of Physics and Astronomy shares this vision. Over the past decade, they have established a fairly sophisticated “sensor Web” infrastructure for the collection and sharing of scientific data. Their system is called RTEMP (Real-Time Environment Monitoring Platform). RTEMP uses Web-based infrastructure to collect longitudinal data from scientific measurement instruments, and share the data with other international researchers [16]. We use the term “scientific Web sites” to refer to such Web sites that host specialized datasets for sharing knowledge with researchers and the general public.

RTEMP currently receives data streams from over a thousand remote Internet-connected devices. The data streams include meteorological data (e.g., temperature, precipitation, wind speed and direction), seismic data, oceanographic data (e.g., water temperature, depth, current, ice thickness and movement), and atmospheric data (e.g., magnetometer readings, auroral images). For devices that generate low-volume telemetry data, the User Datagram Protocol (UDP) is used to relay data to the RTEMP server on a best-effort basis. For devices that generate larger objects, such as photos and infrared images, the Transmission Control Protocol (TCP) is used to deliver data reliably to RTEMP.

A particularly novel aspect of RTEMP’s design is that in addition to collecting data from remote scientific instruments, it also collects meta-data about the operational status of the instruments themselves (e.g., battery power, CPU utilization, available disk space, network connectivity status, system error messages). These status reports are also conveyed using protocol messages, and constitute part of the data streams collected at the server. This meta-data facilitates remote monitoring of the instruments via a dashboard control system, reducing staff time and travel costs for maintenance of the remote equipment.

The University of Calgary hosts several scientific Web sites to share the measurement data from RTEMP’s remote sensors for atmospheric and environmental monitoring. After assessing all the incoming and outgoing network traffic at the University of Calgary, we found that one scientific Web site, namely the *Auroral Imaging Group* (Aurora) site [17], was the top site generating outbound network traffic. This site is hosted within the Department of Physics and Astronomy at the University of Calgary.

The Aurora site studies the aurora borealis (Northern Lights), which is a natural phenomenon caused by cosmic rays, solar wind, and magnetospheric plasma interacting with the upper atmosphere [18]. Since the aurora are mainly observed at night in remote areas, the RTEMP researchers have deployed specially-designed digital and infrared cameras across northern Canada as a ground-based observatory to record auroral phenomena. The Aurora site is a scientific Web site sharing the auroral data collected from these cameras.

The research team in Physics and Astronomy has an ambitious plan to expand the usage of RTEMP in the future. Their intent is to further increase the number and type of sensors, to increase both the sampling frequency (e.g., every 1 s, instead of every 10 s) and resolution for their images, and to support even more users, including school children doing “citizen science” projects. As part of ongoing research collaborations, we were asked to do a capacity planning study to assess the scalability and performance of the RTEMP system design.

There are two primary infrastructure components to RTEMP. One is the back-end server infrastructure for the collection and analysis of sensor data. The other is the front-end Web server for sharing datasets and images with the research community. We studied both of these components in our work, and identified performance issues in each that limit the scalability of the system.

The key contributions of our work are the practical lessons learned from our sensor Web case study. Within RTEMP’s data collection system, we identified a problem with memory management for large TCP data streams. Fixing this issue increased peak system throughput by about an order of magnitude, and also improved system scalability for multi-core operation in the future. For Aurora’s data dissemination, we identified problems with excessive Web robot traffic, as well as Javascript cache-busting techniques. We proposed and evaluated better solutions for data sharing. While our results are based on one particular sensor Web system, we believe that the lessons learned apply more broadly to other education, research, and scientific Web sites sharing remote sensing data [10,19,20].

The rest of this article is organized as follows. Section 2 discusses relevant prior work on sensor Webs and Web traffic characterization. Section 3 presents our evaluation of the data collection side of RTEMP. Section 4 provides our in-depth look at data dissemination aspects of the Aurora Web site. Section 5 summarizes our contributions.

## 2. Related Work

From its inception, the World Wide Web was envisioned as a means of sharing information and research datasets with scientists all around the globe [21]. This information sharing has taken many forms over the past 25 years, including static HTML content, dynamic content, user-generated content, Web 2.0, and more [22,23,24]. A recent trend has been toward “sensor Webs”, also known as the “Internet of Things” [25]. Such a Web provides a highly-connected infrastructure with sufficient information at your fingertips to do decision-making or real-time control, whether in an industrial setting or for environmental monitoring [26]. The ultimate example of a highly-connected data-generating infrastructure is the Square Kilometer Array (SKA) [20]. While the RTEMP project pales in comparison to SKA, it shares many of the same design challenges for real-time processing of incoming data streams.

There is a wealth of literature on Web workload characterization dating from the mid-1990s to the present. This work spans Web client characterization [27], Web servers [28], and Web proxies [29]. Arlitt et al. [28] identified ten common properties in Web server workloads. Crovella et al. [23] discovered self-similarity in World Wide Web (WWW) traffic, while Sedayao et al. [30] analyzed WWW traffic patterns from a fundamental perspective.

More recent Web workload studies have provided longitudinal views of Web workload evolution [31] and modern Web traffic [32]. Many recent studies focus on traffic of Web 2.0 sites, in which users can interact and collaborate with each other instead of merely viewing the content provided by the Web site. Butkiewicz et al. [33] studied the complexity of today’s Web pages. Schneider et al. [24] presented a study of AJAX traffic by analyzing popular Web 2.0 sites, such as Google Maps, and social network Web sites. Eldin et al. [19] studied the top 500 popular pages in Wikimedia, while Urdaneta et al. [34] studied the network traffic for Wikipedia.

There is not much literature about scientific Web sites. For example, Morais et al. [35] studied the user behavior in a citizen science project, though their focus was on user interaction, rather than Internet traffic workloads. Perhaps the closest example in the prior literature is Faber et al. [36]. They studied the Web traffic of four different data sets and compared the workload characteristics to those in [28]. However, their analyses are limited due to missing HTTP header fields in their logs, and a relatively short observation period of 1–2 months. Furthermore, their data was collected over a decade ago, and scientific Web workloads may have changed since then.

Unlike most studies which focus on the outbound request traffic (e.g., studying the viewing pattern of Google, Facebook, and YouTube, from the perspective of a campus network), our work primarily studies the inbound request traffic. Apart from the Aurora scientific Web site, we also found a relatively large volume of traffic triggered by an educational Web site [10], whose traffic is dominated by recorded course videos.

## 3. Data Collection Issues for RTEMP

### 3.1. Problem Statement

RTEMP is intended to support a growing suite of future real-time environmental monitoring projects. If this ambitious goal is to be realized, then the performance limitations of the current system need to be overcome.

The specific research questions of interest were the following:

- How well is RTEMP working in its current configuration?
- What are its performance bottlenecks, if any?
- How can the scalability of RTEMP be improved?

A major concern in RTEMP is that the system receiving the data packets from sensors is a bottleneck, which limits the scalability of the system. Through testing and code analysis, we identified the root cause of its low throughput. This issue is discussed further in Section 3.5.

Other issues were identified as well. First, the software was written by auroral researchers with minimal computer science background. As such, there may be more efficient ways of doing things than the original programmers considered. Second, the system has been running for years. There have been many modifications to the system, to extend functionality beyond the original design. Last but not least, the system was designed to run on a single server, and thus had no fail-over or load balancing mechanisms. These issues limit system scalability and reliability.

### 3.2. System Architecture

The first step to developing a better system begins with understanding the existing solution: what it does and how it does it. The two key components of the system are the external and internal network operations.

Externally, the system listens on specific ports for packets from field devices in operation. When the system receives these packets, it saves them to a folder based on the sender’s Internet Protocol (IP) address, the port used, and the date the file was received. If there is already a file in that folder, the new data is appended to it. The packets contain no information for direct use by the receiving program itself. The data is simply indexed and stored as quickly as possible for later use. There is no response provided beyond standard TCP interactions.

Internally, the program that listens for and receives packets from the field devices runs independently of the rest of the software in the RTEMP backend. The data it saves to the storage server contains the necessary information for other programs to process the data, and make it Web accessible.

### 3.3. Design Goals

There were four main design goals for the new system. First, it should be “plug and play”. The new code must preserve all previous functionality, to be fully backwards-compatible with the old system. Second, the new solution must be scalable to effectively utilize more powerful systems, including multi-core or multi-server systems. Third, the new system should allow multiple machines to dynamically share the receive load, and prevent downtime or data loss due to individual machines being removed for maintenance. Fourth, the code should be more modular. Documentation and readability are needed to ease modification and debugging in the future.

### 3.4. Experimental Methodology

In order to test the performance of the RTEMP software without disrupting system operation, a separate test environment was set up with two computers connected through a 1 Gbps LAN. The first computer hosts one virtual machine that runs either the old version of the RTEMP server (receive side) code, or the new version, depending on which test is being run. The second computer hosts three virtual machines that each run a copy of the client (send side) code that has been modified for testing. Both test computers were similar, but not identical, to the high-end Linux system used to host the RTEMP platform.

This test environment was used throughout the development of the new software. As the tests were run, the virtual machine hosting the server was modified to allow it to run on varying numbers of cores. Similarly, the number of sending Virtual Machines (VMs) was modified, and each test involved a range of file sizes typical of the RTEMP system (32 KB to 1 MB), plus some anticipated in the future (8 MB).

Table 1 summarizes the factors used in our benchmarking experiments. A full-factorial experimental design was used (i.e., all combinations of factors and levels). Each experiment ran for 2 min, with the average results reported.

The primary performance metric of interest in the experiments reported here is TCP throughput, expressed in Megabits per second (Mbps). We are particularly interested in how aggregate throughput varies based on the number of sending clients, the number of server cores, and the file size.

### 3.5. Experimental Results

**Figure 1:**
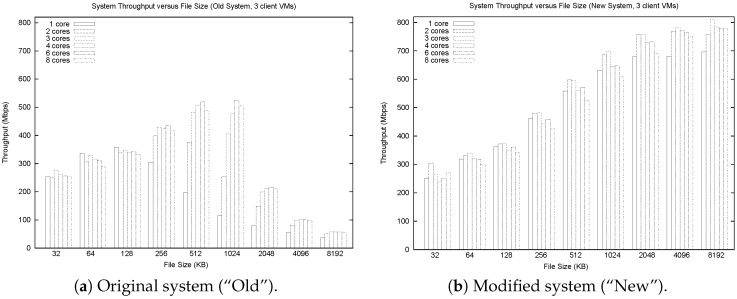
RTEMP system throughput versus file size.

Figure 1 highlights the main results from our study. The graph on the left is for the old system, while that on the right is for the new system. These graphs show TCP throughput on the vertical axis, with file size on the horizontal axis (log scale). Each cluster of six bars shows results for different numbers of cores, ranging from 1 to 8 (left to right).

The main observation from Figure 1 is that TCP throughput increases steadily at first with file size in the old system, but drops off sharply beyond file sizes of 1 MB. This behavior guided us to a deeper investigation of the TCP stream handling code [37]. Another clue was the dramatic performance difference between 1-core and 4-core performance on 1 MB files. In particular, this indicates a CPU-intensive workload, since the throughput performance for 1 core actually peaks at 128 KB, and it is only the presence of extra cores that sustains the throughput for 1 MB files.

The root cause of the observed throughput drop was inefficient memory management in the receive side code for TCP stream handling. In particular, the receiver would “grow” the memory space allocated for an object by one packet’s worth of data at a time. Worse yet, it would do so by allocating new memory, copying all the old data from the previous memory location, and de-allocating the previous space. This naive implementation caused the system to perform an excessive number of memory allocations (i.e., O(*n*) in the number of packets received for an object), and excessive data copying (i.e., O($n^{2}$) in the number of packets received for an object).

In our revised implementation, we considered both static and dynamic approaches to memory management. The simplest approach statically allocates 8 MB per TCP data stream received. This requires only one memory allocation and only a single copy for each piece of incoming data. The performance of this system is shown on the right in Figure 1. Throughputs as high as 800 Mbps are achieved on a resource-rich machine. We also implemented a threshold-based approach that starts at a more conservative 128 KB, and quadruples the memory allocation whenever the allocated space fills up. As expected, this approach (not shown) is slightly slower than the static allocation approach on large files, since up to three extra data copies may be required.

**Figure 2:**
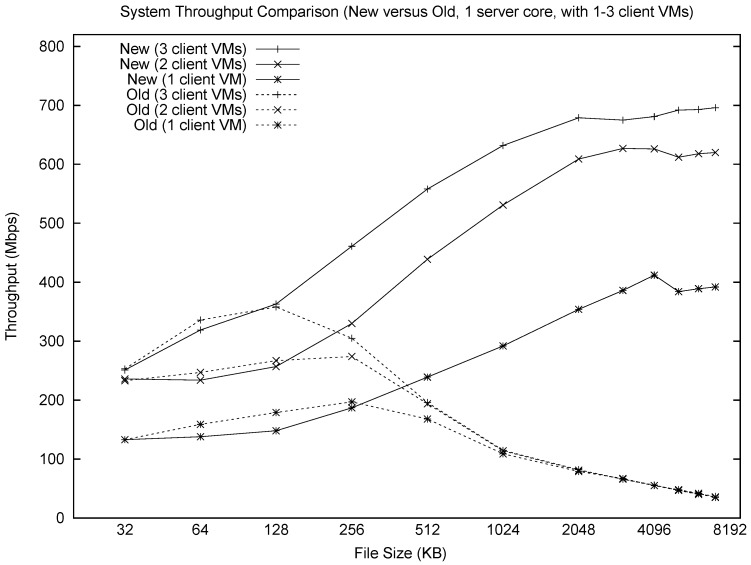
Throughput comparison between original (“Old”) and modified (“New”) system.

Figure 2 shows a summary comparison of the new and old systems, as a function of file size and number of sender VMs. The revised RTEMP system shows much more stable and scalable performance than the old system; even a single core can achieve a throughput of 700 Mbps. The performance differences are negligible for small files (e.g., 128 KB), but the throughput differs by up to a factor of 12 on 4 MB files.

### 3.6. Summary

By finding and fixing a major issue with the server’s memory management, the new system is not only up to 12× faster than the old system, but it is more efficient as well. While most of the performance gains occur for larger files, the improved multi-core efficiency is evident throughout the range.

In addition to the performance and efficiency gains, the new server side code has been modularized, documented, and designed for future expansion. The client side code has also seen significant improvements that allow it to dynamically load balance across server ports, and to automatically failover to another server if the one they are sending to goes down. Both the server side code and the client side code are fully backwards compatible with the old system, which should make future system upgrades straightforward.

## 4. Data Dissemination Issues for Aurora

Based on our measurements of the campus network traffic, we observed that the traffic generated by the Aurora site is relatively large. Specifically, we observed about 1.5 million HTTP requests sent to the Aurora server daily, to retrieve about 90 GB of data volume each day.

These observations led to three specific research questions:

- How is the Aurora site being used by its community?
- Why is the Web traffic volume so high?
- How can the operation of the site be improved, without compromising functionality for its users?

The primary motivation for our study is a better understanding of the usage of the Aurora Web site in particular, and scientific Web sites in general. Considering the traffic volume triggered by the Aurora site, we want to measure its network traffic, identify performance issues (if any), and propose potential remedies for the problems. These issues are particularly germane as the Aurora group contemplates the design of its next-generation “sensor Web” infrastructure, with additional sensors, higher-resolution images, and finer-grain temporal sampling.

### 4.1. Methodology

We assessed the performance of the Aurora Web site through a Web workload characterization study, using passive network traffic measurement. At the University of Calgary, all incoming and outgoing Internet traffic passes through the edge routers on the campus backbone. By mirroring these traffic flows to a network traffic monitor, we record all the packet-level headers between the campus and the Internet. We use a monitor server (Dell, 2 Intel Xeon E5-2690 CPUs, 64 GB RAM, 5.5 TB storage, CentOS 6.6 × 64) with an Endace DAG 8.1SX card equipped for traffic capture and filtering. The Endace DAG card is designed for 10 Gbps Ethernet, and uses a series of programmable hardware-based functions to improve the packet processing performance. A Bro network security monitor [38] is deployed to extract and record TCP/IP traffic from the filtered traffic streams.

Once the logging system is activated, Bro collects and generates logs hourly. The Aurora site studied in our work is an HTTP server. Therefore, we concentrate on HTTP traffic measurements. The HTTP transaction logs contain detailed information about the request and response headers, including request method, referer, user agent, and response code.

We analyze the traffic logs for a four-month period from 1 January 2015 to 29 April 2015. We focus solely on the HTTP traffic of the Aurora site, particularly inbound HTTP requests and outbound data volume.

### 4.2. Passive Measurements: Workload

We conducted a workload characterization study, using passive network traffic measurements, to help understand how the Aurora site is being used by its research community and the general public.

The Aurora site is hosted and maintained by the Auroral Imaging Group (AIG). They use 60 ground-based all-sky cameras to take carefully-synchronized images of the arctic night sky, to study auroral phenomena and the magnetosphere. Images from the remote cameras are sent to the Real-Time Environment Monitoring Platform (RTEMP) [16] at the University of Calgary. These images are made available publicly on the Aurora site, which is also accessible via the AuroraMAX portal [39] on the Canadian Space Agency (CSA) Web site.

The Aurora group is part of a larger collaborative project called THEMIS [40], which is based at the University of California at Berkeley (UCB). The UCB researchers regularly retrieve the images from the Aurora site, and archive them at their own institution. Other THEMIS collaborators include the University of Alaska, the University of Alberta, and Athabasca University.

**Figure 3:**
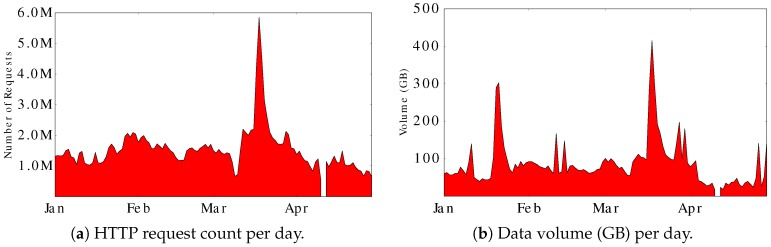
HTTP requests and data volume per day.

Table 2 provides a statistical summary of the HTTP traffic over the four-month period under study. Figure 3 shows the daily count of HTTP requests for the Aurora site as well as outbound traffic volume. The Aurora site had fairly steady request traffic throughout most of the observation period (except for a brief monitor outage on 11 April), but with a surge near 6 million requests per day in mid-March 2015. The latter surge is related to geo-magnetic storm activity affecting the aurora (see Section 4.2.6).

#### 4.2.1. IP Analysis

**Figure 4:**
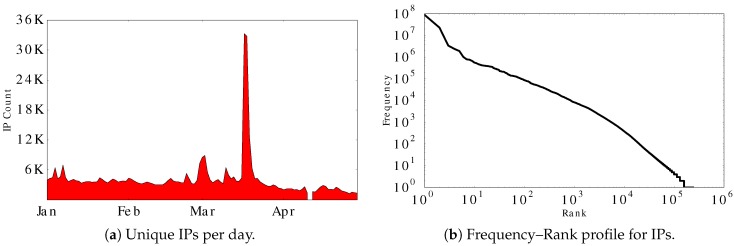
Statistical information for IP addresses observed.

There were 240,236 distinct IP addresses that visited the Aurora Web site during our trace. Figure 4a shows the number of distinct IP addresses per day. The daily count for unique IPs is about 4000, except for mid-March, when the IP count grows eightfold. Figure 4b shows the frequency–rank profile for the IP addresses observed at the Aurora site. The linear trend on this log–log plot provides visual evidence of power-law structure in the frequency distribution, which is often seen in human access patterns to information systems [41]. Least-squares regression confirms a strong linear fit, with slope value −1.88 and $R^{2}$ value of 0.99.

We performed IP geolocation for all the IP addresses, using the IP location services from IPAddressLabs [42] and MaxMind [43]. The IP addresses came from 192 distinct countries in total. Table 3 shows the IP geolocation distribution of the top five countries, which include Canada and the United States as the top two countries. Since the THEMIS project is based in North America, these results are not surprising. Many of the IPs (39.5%) are from Canada, with the United States second at 15.7%. However, most of the requests (73.2%) come from the United States, with Canada second at 17.5%. Clearly, the Web request patterns from these two countries differ greatly.

Table 4 shows the geolocation information for the top five most frequently observed IP addresses, ranked by number of HTTP requests. Three observations are evident from these results. First, most of the top five are members of the THEMIS project, as expected (e.g., University of California at Berkeley, University of Alaska). Second, some of these organizations have multiple IPs, indicating either multiple auroral researchers, the use of automated robots, or the use of DHCP (Dynamic Host Configuration Protocol). Third, the topmost IP address, which is from UCB, generates about half of the requests. Its total request count dominates traffic, both on a daily basis and overall.

#### 4.2.2. HTTP Requests and Responses

The HTTP request–response statistical results are shown in Table 5. About 88% of the HTTP requests use the GET method, while 12% are HEAD requests. Among the HEAD requests, over 99.7% are generated by Wget. Other HTTP methods are negligible with fewer than 100 requests over the four-month period. For responses, 95.59% are “200 OK”, and about 2% are “304 Not Modified”.

#### 4.2.3. URL Analysis

There were 2,894,294 unique URLs visited during our trace. (Actually, the number of unique URLs observed is 75,847,177. When users fetch data (mostly images and videos) from the Aurora site for live updates, they append a timestamp to the URL as a query string to obtain fresh content. For example, the request URL “/abc/latest.jpg” is modified to “/abc/latest.jpg?1426417182905” by the JavaScript code. This “cache busting” technique inflates the number of unique URLs.) The most frequently requested URLs are images or videos labeled with recent or latest in the “/summary_plots/” directory. These images are updated regularly by the ground-based cameras every night, while the videos are generated the next day. The topmost URL for the Yellowknife location “/summary_plots/slr-rt/yknf/recent_480p.jpg” accounts for 18% of the requests and 40% of the data volume.

There are 39 different file types observed in our trace. JPEG images account for most of the requests (52.23%) and data volume (72.50%). Static HTML files are popular in terms of requests (36.80%), but contribute little to data volume (1.35%). A similar observation applies for JavaScript files.

**Figure 5:**
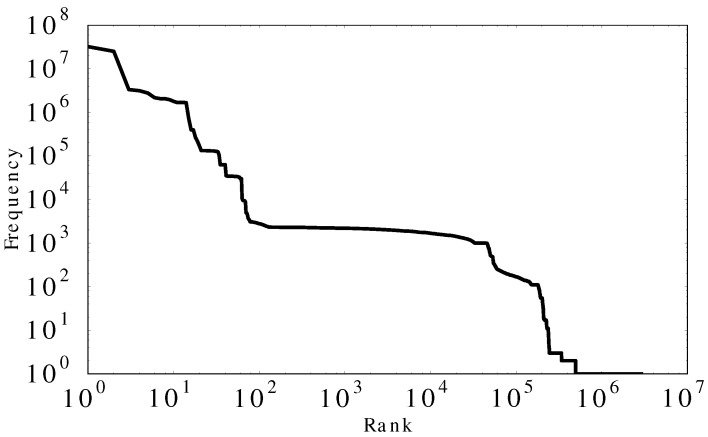
Frequency–Rank profile for URLs.

Figure 5 shows a frequency–rank analysis applied to the URLs requested on the Aurora site. It has several distinct plateaus in the frequency–rank profile. This frequency distribution does not fit the power-law structure very well with slope value −1.23 and $R^{2}$ value of 0.72. We attribute this to machine-generated request traffic, which we explore in more detail in Section 4.2.5.

#### 4.2.4. HTTP Referer

The HTTP referer field (when present) in the HTTP request header indicates the Web page from which the Aurora site was visited. We analyze the top 100 referers in terms of requests and data volume. The top referer for both is the CSA AuroraMAX portal, which appeared in 45,763,205 requests (25%), and triggered a total data transfer volume of 4423 GB (43%). Most of the referrals come from pages showcasing images or videos from the Aurora Web site. For example, 25 of the top 100 referrers come from the CSA site, and nine from virmalised.ee [44], which is an Estonian Web site broadcasting live auroral imagery from cameras around the world. These live feed pages generate large volumes of network traffic. Interestingly, many of the referring Web pages use the JavaScript “cache busting” technique to refresh the images every few seconds, contributing to the machine-generated traffic. (Note that the browser will refresh the image automatically, whether there is a human viewing the images or not.) The naive approach used by these referer sites for fetching images is not very network-efficient.

#### 4.2.5. Robot Traffic

We next explore our second research question, regarding why the request traffic volumes are so high. The primary reason is the predominance of automated Web crawling robots in the traffic.

The top four IPs from University of California at Berkeley (UCB) and University of Alaska (UA) in Table 4 are robots. We use the terms “UCB1” and “UCB2” to refer to the two most prominent IPs from UCB. Similarly, we use “UA1” and “UA2” to refer to the two most prominent IPs from UA.

**Figure 6:**
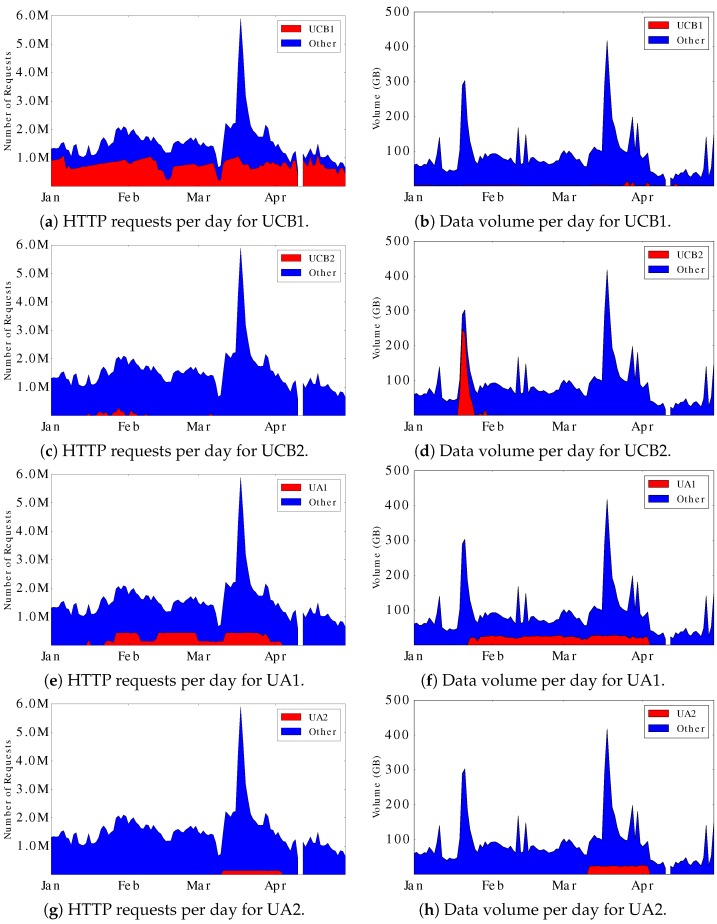
HTTP requests and data volume per day for UCB and UA Web robots.

Table 4 summarizes the HTTP requests and data volume information for the four robots. Figure 6 shows the daily HTTP requests and data volume information for UCB and UA robots. In these graphs, the blue curves show the overall traffic for the Aurora site, while the red curves show the request traffic and data volumes for the four individual robots, each of which has different behaviors.

Our analysis leads to the following four observations:

1. UCB1 generates 89,977,861 requests in total. This is about half of the total Aurora request traffic. However, the daily data volume that UCB1 generated is comparatively small. Upon further analysis, we found that all the requests generated by UCB1 have the user agent Wget/1.11.4 Red Hat modified. This is a software tool for retrieving files from a Web site, and recreating a copy of the site structure.
2. UCB2 was active primarily from mid-January to early-February. It only generated 1,919,161 requests in total. Nevertheless, it contributed much of the data volume in late-January. Unlike UCB1, it uses another version of Wget, namely Wget/1.12 (linux-gnu), as the user agent.
3. UA1 generated approximately 0.2 million requests and 14 GB of data volume per day. It has more impact on data volume than the other robots.
4. UA2 was active in March, when the geo-magnetic storm happened.

Further analysis of the robots’ workload patterns show that:

- UCB1 runs two independent Wget scripts. Both scripts use recursive download mode to save all files in the given directory to the local hard disk (it downloads HTML files but deletes them after extracting any embedded URLs). The “imager” robot updates local data from imager/stream1, imager/stream2, and imager/stream3 directories. It usually takes 2–3 h to complete the scan of a month’s data, and 4–6 h in total to complete both last month and the current month. The “fluxgate” robot updates local data from the fluxgate/stream0 directory. It usually takes 10 min to retrieve 2 months of data. Both robots take a short break after one complete scan over 2 months of data. The length of the break between scans varies from around 1 h to 4 h. The robots use time-stamping mode, which makes Wget send HEAD requests to check the time-stamp of files on the server-side and only generate GET requests to fetch a file if it has a newer time-stamp. It is inefficient that UCB1 runs the Wget scripts several times each day, even though the content rarely changes. Furthermore, it does so using HTTP/1.0 (non-persistent connections by default), and many HEAD requests, rather than Conditional GETs. In contrast to a persistent connection, in which the HTTP connection reuses the existing TCP connection, the non-persistent HTTP approach establishes a new TCP connection for each request–response transaction. This approach adds extra overhead for the Web server, because of the repeated handshaking for setup and termination of short-lived TCP connections. It also increases the response time for HTTP transactions, because of the extra network round-trip times incurred, and the use of slow-start for TCP congestion control. A persistent-connection approach could substantially reduce data retrieval times, particularly over a WAN.
- UCB2 uses Wget/1.12 (linux-gnu) to retrieve files in older directories. Different from UCB1, the UCB2 script generates the URLs itself and invokes Wget to directly fetch the files.
- UA1 and UA2 robots are browsers that repeatedly request the RTEMP live feed pages. Similar to the CSA AuroraMAX page, RTEMP provides Aurora live feeds by re-fetching images and videos from the Aurora site server. The process is completed every three seconds by the client’s browser, implemented with JavaScript DOM operations. The two UA robots did the same task, both re-fetching the live-feed images regularly for months.

#### 4.2.6. Geomagnetic Storm

An interesting discovery in our dataset was the non-stationary traffic observed in mid-March 2015. The HTTP request traffic and the data volume both quadrupled from their normal levels for the 17–20 March period (see Figure 3).

The root cause for this traffic surge was solar flare activity that triggered one of the largest geomagnetic storms in over a decade [45]. Auroral researchers knew about this event, and eagerly downloaded many of the new images. The ensuing media coverage of the geomagnetic storm triggered many other site visits, either directly or via the AuroraMAX portal. Further analysis indicates that the increased traffic is primarily human-initiated, since: (1) the number of distinct IPs visiting the site surged eightfold during the geomagnetic storm period (Figure 4a); (2) the number of GET requests quadrupled in the surge, with no change for HEAD requests; and (3) there was a ten-fold increase in the AuroraMAX portal traffic (requests and data volume) during this period.

Our network traffic analysis shows how real-world events can affect the traffic of scientific Web sites. The traffic information shows that flash crowds are not limited to “popular Web sites”. Such surges are important to consider when designing scientific Web sites, especially when provisioning server-side capacity configurations.

### 4.3. Active Measurements: Network Efficiency

Our passive measurement reveals several network inefficiency issues for the Wget robots and the “cache busting” technique adopted by the referer sites. In this section, we propose and evaluate possible solutions for these inefficiencies using active measurements. These solutions address our third and final research question, by providing practical advice for improving scientific Web sites.

#### 4.3.1. File Transfer Methods

To evaluate file transfer methods, an active measurement experiment was performed both in a LAN test environment and a SoftLayer [46] cloud-based WAN environment. A simple Node.js Ecstatic Web server [47] with a subset of the Aurora site content (37,655 files, 53 MB in total) was deployed server-side, and client-side software tools were launched to fetch files from the server. The Node.js server serves static files, mimicking the functions provided by the Aurora server.

In our experiment, we consider software tools that support recursive file transfers and modified-file transfer only. Table 6 provides a summary of our selected methods for file transfer and/or synchronization between multiple sites.

Table 6 shows the results from our experiments. In “Initial Copy”, the software tools are invoked to retrieve files in the “data/themis/fluxgate/stream2/2015/” folder (1 January to 27 April) from our server. In “Subsequent Copy”, the data of 28 April (four new data files) are added to the server and all software tools are executed again. The number of files, data volume, and elapsed time are recorded for the two file transfer experiments.

From the Table 6 results, and the server-side logs, we find:

- Wget supports FTP and HTTP(S), which is convenient and flexible. However, it takes a long time to retrieve all the files, downloading additional HTML files for extracting the file directory information (this is slow when each HTML page has many links). For the LAN test, there is a small difference in time consumption for the three different versions of Wget. Wget 1.11.4 is a little slower than version 1.13 and 1.16. Wget 1.11.4 does not support HTTP/1.1, so some of its requests result in closed connections. Note that Wget 1.13 is the first released version supporting HTTP/1.1 [48]. Wget 1.11.4 supports “Keep-Alive" connections with HTTP/1.0, if the server side permits it. For the WAN test, the time consumption for Wget 1.11.4 and Wget 1.16 are about the same.
- rsync has the best performance in both LAN and WAN tests, taking less than a minute to download/update all the files. Specifically, it is about five times faster than using Wget 1.11.4 for the initial copy, and about 10 times faster for each subsequent copy. Furthermore, it applies the zlib library [49] to reduce data volume by about a factor of 4 in this case. The drawback is that rsync does not support HTTP. Nevertheless, the server can provide anonymous rsync service with a daemon running in the background. It is interesting that rsync uses less time in the WAN test than the LAN test. Note that rsync adopts a specially designed algorithm to check the differences between files among server-side and client-side, which is CPU-intensive. Therefore, the WAN test may take less time since the cloud server is more powerful than the shared server in the LAN test, even though the WAN itself is inherently slower.
- HTTrack and lftp are worse than Wget, requiring a long time to complete the task. lftp is executed with FTP service, which saves bandwidth for downloading additional HTML files.

#### 4.3.2. JavaScript Cache-Busting Solution

Many referer sites (i.e., AuroraMAX, RTEMP) use “cache-busting” to obtain the latest image from the Aurora server, instead of reading from cache files. We propose an AJAX HEAD implementation for solving this inefficiency problem, with three primary considerations:

1. The site designers want users to be able to view the new images as soon as possible. Therefore, simply increasing the refresh interval is not an appropriate approach for guaranteeing the same user experience.
2. The approach should not increase load on referer Web servers (i.e., a proxy server). Our approach has the browser send HEAD requests every few seconds to check whether the image changed or not. If the image changes, it generates a GET request with “cache-busting” technique to fetch the new image. The image files are still transfered between the Aurora server and the browser.
3. The changes made to the current Aurora server and referer Web pages should be minimal. The AJAX HEAD method only requires the Aurora server to support cross-origin resource sharing (CORS) by adding one line to the Apache server configuration file, which is needed to bypass the same-origin policy.

We performed an active measurement experiment comparing the bandwidth consumption of “cache-busting” with our AJAX HEAD implementation. To be specific, we wrote a Python script to download the latest image from the Aurora server, in a scenario where the image changes every 30 s. We emulate the two methods by rendering two HTML pages in Chrome (v. 45) browser (the result is similar for the latest version of popular browsers, such as Safari and Firefox). In our experiment, there is only one client visiting the “referer site”; in practical application, the bandwidth savings would scale with the number of site visitors.

**Figure 7:**
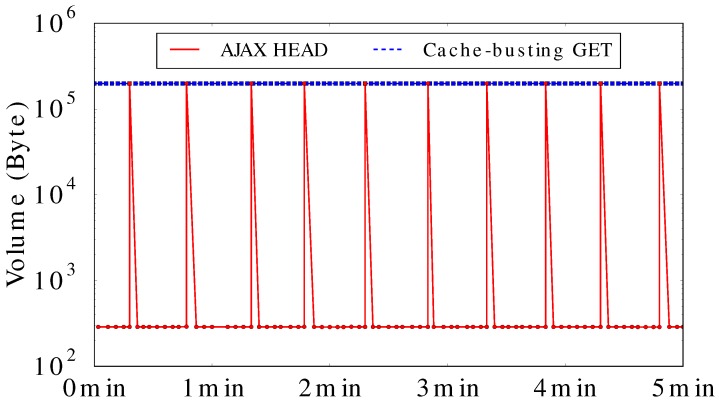
HTTP response size results for the two methods.

Figure 7 shows the HTTP response size of the server-side logs during 5 min of observation. The x-axis is time, while the y-axis is the data volume transferred. The graph shows that the cache-busting GET sends about 200 KB of image data for every request, while the AJAX HEAD method only does so when the image has actually changed. The latter approach returns only about 200 bytes of HTTP header for most of the requests. The AJAX HEAD method dramatically reduces the bandwidth usage, compared to the “cache-busting” approach. Conditional GET is an alternative solution, which enables the client’s browser to GET the latest image if it has been changed, or do nothing if it remains the same.

#### 4.3.3. Summary of Network Efficiency Experiments

In summary, the practical solutions for improving network efficiency are:

- HTTP and rsync. The Aurora server can provide HTTP service to public researchers, and specifically support rsync service for robots crawling the data. We recommend using a newer version of Wget that supports HTTP/1.1 and persistent connections for downloading data files.
- RSS. The Aurora server can inform users regarding what data files have changed, and when, via the RSS service. Bandwidth can be saved by avoiding unnecessary retrievals.
- AJAX HEAD. Instead of using “cache-busting” for retrieving the latest image files, we recommend that referer sites apply the AJAX HEAD implementation for saving bandwidth. Conditional GET is an alternative to the AJAX HEAD method.

### 4.4. Summary of Aurora Issues

We studied the network traffic workload of a scientific Web site over a four-month period. We analyzed the HTTP traffic with passive measurements, and performed active measurements for studying network usage efficiency.

Our main observations are summarized as follows:

- Sensor Web sites can generate relatively large volumes of Internet traffic, even when the user community seems small.
- Robot traffic and real-world events can dramatically increase network resource usage.
- A large fraction of the observed network traffic in the Aurora site can be reduced with more efficient networking solutions. We suggest a separate rsync service for robots, and the AJAX HEAD approach for displaying live-feed images.

## 5. Conclusions

In this article, we studied the performance of the hardware/software infrastructure supporting the RTEMP “sensor Web” platform at the University of Calgary. We focused on both the data collection and the data dissemination components of this system.

Through standard benchmarking techniques, we assessed the scalability, robustness, and failover capability of RTEMP. While we found no major problems with UDP stream handling in RTEMP, we discovered and fixed a serious performance issue with TCP stream handling. This fix improved TCP throughput of the system by about an order of magnitude. In addition to this key fix, we improved the efficiency of RTEMP when running on multi-core servers, implemented a new load balancing mechanism across ports, and added a failover mechanism. Our efforts have helped “future proof” RTEMP for the next few years, with no new hardware investment required.

We also used network traffic measurement for a workload characterization study of the Aurora system, from which three observations emerged. First, the data volumes transferred across our campus network by this site are relatively large, given the relatively small user community. Second, the network traffic is heavily influenced by robot traffic and real-world events, with robots using either Wget for site crawling or JavaScript for browser refresh operations. Third, these approaches seem inefficient for what they are trying to accomplish. Our active measurement study demonstrated more efficient ways to achieve file synchronization and archival across the Internet. Such approaches could make this scientific Web site more scalable and Internet-friendly.

Our work highlights several practical issues related to the design and operation of scientific Web sites that share data publicly with researchers and citizen scientists. While most of our observations are specific to the Aurora site, we believe that some will apply more broadly to other scientific Web sites, particularly those that use automated means to collect and share scientific datasets from remote sensing infrastructure (e.g., Internet of Things, SKA [20]). Improving the design of such sites is important as they increase in number and scale, as well as temporal and spatial resolution. We hope that our results provide useful, practical advice to the designers of scientific Web sites, so that these sites are scalable and Internet-friendly.

## Figures and Tables

**Table 1:** Experimental factors and levels.

Factors	Levels
RTEMP Software Version	{Old, New}
Number of Client VMs	{1, 2, 3}
Number of Server Cores	{1, 2, 3, 4, 6, 8}
File Transfer Size (KB)	{32, 64, 128, 256, 512, …, 8192}

**Table 2:** Statistical characteristics of the aurora site (1 January 2015 to 29 April 2015).

Total Reqs	Avg Reqs/day	Total GB	Avg GB/day	Uniq URLs	Uniq IPs
182,068,131	1,529,984	10,354	87.01	2,894,294	240,236

**Table 3:** IP geolocation distribution, top five countries.

Country (by IPs)	IP	Pct.		Country (by Reqs)	Reqs	Pct.
Canada	94,942	39.5%		United States	133,941,542	73.2%
United States	37,669	15.7%		Canada	31,952,429	17.5%
United Kingdom	15,139	6.3%		Japan	4,587,971	2.5%
Germany	10,985	4.6%		Germany	2,378,523	1.3%
France	5730	2.4%		United Kingdom	1,755,945	1.0%

**Table 4:** Top five most frequently observed IP addresses.

IP	Reqs	Pct.	Total GB	Pct.	Organization
128.32.18.45 (UCB1)	89,977,861	49.4%	211	2.0%	University of California, Berkeley, CA, USA
137.229.18.201 (UA1)	22,951,449	12.6%	1680	16.2%	University of Alaska, Fairbanks, AL, USA
137.229.18.252 (UA2)	3,403,550	1.9%	573	5.5%	University of Alaska, Fairbanks, AL, USA
50.65.108.252 (Shaw)	2,394,630	1.3%	34	0.3%	Shaw Communications, Edmonton, AB, Canada
128.32.18.192 (UCB2)	1,919,161	1.1%	789	7.6%	University of California, Berkeley, CA, USA

**Table:** 

HTTP method	Pct.
GET	88.4%
HEAD	11.6%

**Table:** 

HTTP status code	Pct.
200 OK	95.59%
206 Partial Content	0.14%
304 Not Modified	1.99%
404 Not Found	0.44%
Other	1.84%

**Table 6:** Experimental results for different file transfer/synchronization methods.

Software tool	Initial copy of files			Subsequent copy of files		
	Files	Volume	Time	Files	Volume	Time
Wget (v. 1.11.4)	37,782	62 MB	5 min 13 s	132	9.9 MB	2 min 49 s
Wget (v. 1.13)	37,782	62 MB	5 min 1 s	132	9.9 MB	2 min 41 s
Wget (v. 1.16)	37,782	62 MB	4 min 52 s	132	9.9 MB	2 min 29 s
rsync (v. 3.1.1)	37,655	16 MB	49.8 s	4	873 KB	14.6 s
lftp (v. 4.6.0)	37,655	53 MB	27 min 58 s	4	316 KB	2 min 9 s
HTTrack (v. 3.48-19)	37,782	62 MB	2 h 52 min	132	19 MB	1 h 18 min
Remote file transfer between campus network and cloud service						
Wget (v. 1.11.4)	37,782	62 MB	1 h 3 min	132	9.9 MB	29 min 32 s
Wget (v. 1.16)	37,782	62 MB	1 h 2 min	132	9.9 MB	31 min 22 s
rsync (v. 3.1.1)	37,655	16 MB	23.3 s	4	873 KB	5.4 s
